# Metastasis of Breast Lobular Carcinoma to Endometrium Presenting as Recurrent Abnormal Uterine Bleeding: A Case Report and Review of Literature

**DOI:** 10.1155/2019/5357194

**Published:** 2019-02-24

**Authors:** Rodolfo Franco-Márquez, Adrián Gerardo Torres-Gaytán, Marcela Anahí Narro-Martinez, Anahí Carrasco-Chapa, Benjamín Gómez Núñez, Estefanía Boland-Rodriguez

**Affiliations:** ^1^Pathology Department, University Hospital “Dr. Jose E. Gonzalez” and Medical School of the Autonomous University of Nuevo Leon, Francisco I. Madero and Gonzalitos, 64460 Monterrey, NL, Mexico; ^2^Surgery Department, University Hospital “Dr. Jose E. Gonzalez” and Medical School of the Autonomous University of Nuevo Leon, Francisco I. Madero and Gonzalitos, 64460 Monterrey, NL, Mexico; ^3^Medical School of the Autonomous University of Nuevo Leon, Francisco I. Madero and Gonzalitos, 64460 Monterrey, NL, Mexico

## Abstract

There are few reports of breast cancer cases with uterine metastases. Here, we report a metastatic lobular carcinoma to endometrium presenting as abnormal uterine bleeding. Diagnosis was based in previous lobular breast carcinoma and immunohistochemistry.

## 1. Background

Breast cancer is known for present metastases frequently in advanced stages; the most common sites of metastasis are the liver, bone, and lungs [1, 2].

Metastases to the uterus due to extragenital cancer are rare [3]; it is reported that around 8% of breast carcinomas metastasize to the uterus [4]. Endometrial metastases rarely have origin in a primary extragenital tumor, with an incidence of 4.7%. Literature reports 3.8% of breast tumors metastasize to the uterus. Invasive lobular breast cancer comprises 10% of the invasive types of breast cancer, which is the second in frequency after the infiltrating ductal type [5]. Lobular carcinoma is the most common type of breast carcinoma that metastasizes to the uterus [3]. 60% of patients with invasive lobular carcinoma have metastases at the time of diagnosis [6]; usually the metastases of this subtype of breast cancer are peritoneum, ovary, and gastrointestinal tract [7].

Here, we report a case of invasive lobular carcinoma with endometrial metastases.

## 2. Case Report

An 86-year-old Mexican postmenopausal woman with no remarkable family history of cancer was referred to a gynecologic consult. As a pathological personal history of importance she has medical history of hypertension since she had 40 years old. A previous diagnosis of lobular cancer of the left breast (30 years ago) was made and treated with radical breast mastectomy associated with unilateral axillary lymph node dissection. The pathology report was consistent with lobular carcinoma with no positive lymph nodes and ER/PR+ and Her-2Neu+. After surgery, she received adjuvant chemotherapy with no data about available.

She came to our hospital with a 3-month complaint of abnormal uterine bleeding.

As part of the approach, a transvaginal US was performed and everything was under normal parameters, except for an endometrial thickness of 15mm with heterogeneous echogenicity; however it had no focal thickening; the US was not conclusive for any diagnosis. Therefore the patient underwent to a dilatation and curettage biopsy to take an endometrial biopsy for tissue diagnosis.

Microscopic examination revealed epithelial cells infiltrating the endometrial stroma in certain areas; normal endometrial stroma was also identified.

**Figure 1:**
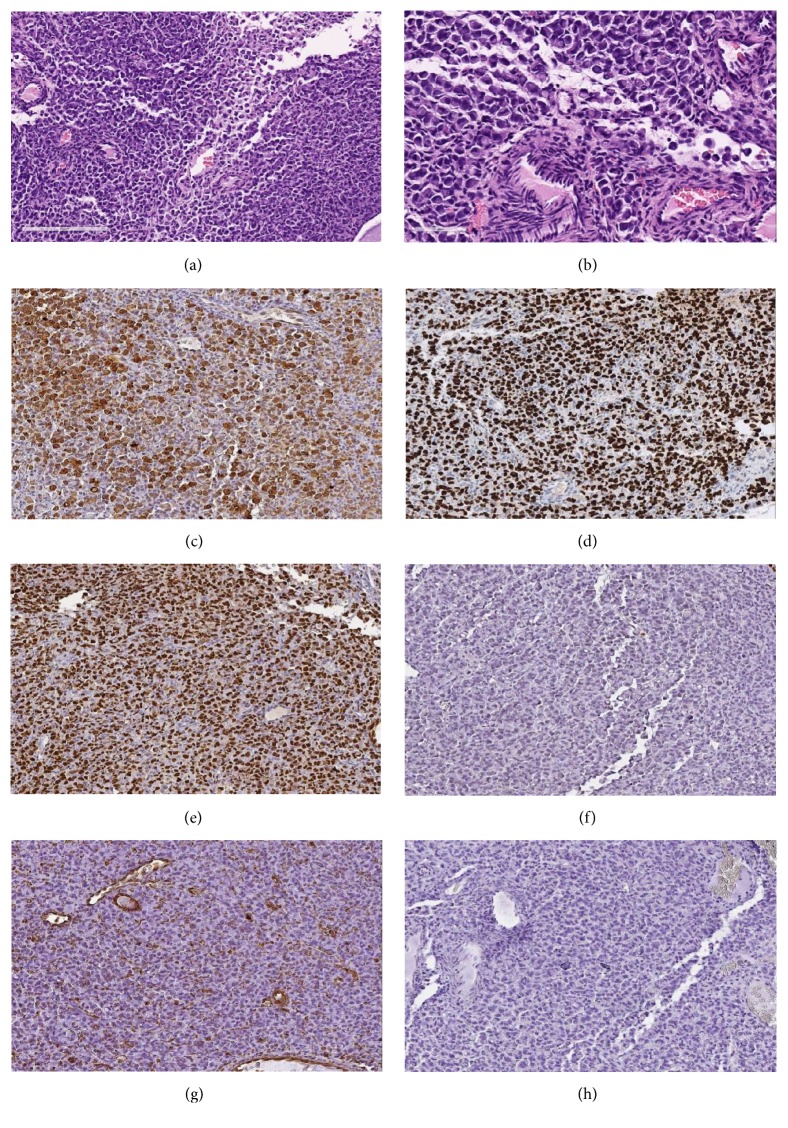
*Pathological microscopic examination*. (a-b) 20X-40x. Hematoxylin-Eosin shows replacement of endometrial stroma by tumor cells, solid architecture with infiltrative growth as single cells was shown, and discohesive cells are observed with plasmacytoid and “signet ring” morphology with scant cytoplasm and rejected hyperchromatic nucleus. (c) 20x. Mammaglobin immunohistochemistry staining shows positivity in malignant cells. (d) GATA3 immunohistochemistry staining shows strong nuclear positivity in malignant cells. (e) 20x estrogen receptor immunohistochemistry staining shows strong nuclear positivity. (f) 20x PAX-8 immunohistochemistry was negative. (g) 20x vimentin immunohistochemistry staining shows positivity just in blood vessels but it is negative in malignant cells. (h) CDX-2 immunohistochemistry staining shows negativity discarding an origin in digestive tract.

The first diagnostic impression was a metastasis from primary cancer in the patient, to corroborate it immunohistochemistry staining (IHC) were requested and endometrial specimens were positive for GATA-3, ER and mammaglobin antigens and negative for PAX-8, CDX-2, and vimentin (Figure 1). The positivity for these markers indicated us and supported the diagnosis of lobular breast carcinoma metastasis to endometrium. The patient was referred to oncology for treatment.

## 3. Discussion

Lobular breast carcinoma usually appears between 60 and 70 years of age [7]. The most common location of this type of tumor is the upper external quadrant of the breast [6].

In a study from Turkey, it was reported that the ovaries are the most affected metastatic organs with 75.8% affectation, followed by vagina (13.4%), uterus (4.7%), cervix (3.4%), vulva (2%), and uterine tube (0.7%) [8].

We found our patient in a prevalence group of around 4.7% (11) [8].

It has been postulated that, due to the characteristics of the ovarian stroma (Ph, oxygen, and tension), it provides a greater susceptibility to be site of implantation and development of metastasis, in contrast to the cervix or endometrium.

The case of metastatic disease of the cervix presents an inflammatory cellular response with fibrous proliferation that conditions an expanded indurated cervix [9].

Few studies have discussed breast metastases to the uterus. It is known that 80% of metastases to gynecological organs are due to a primary breast cancer. The reason between the difference in the metastatic pattern of ductal and lobular infiltration is still not clear, it has been postulated that the expression of cellular adhesion molecule E-cadherin in inflammatory lobular carcinoma, but not in ductal carcinoma, could be related to different metastatic disseminations. Kowalski et al. had found different expression patterns of E-cadherin membranous and cytoplasmic (normal or aberrant) between lobular carcinoma and infiltrating ductal at the primary and metastatic site, suggesting that this protein plays a different role in the evolution of each tumor [10].

Another study revealed that the most common site of metastasis within the uterus is myometrium and that abnormal vaginal bleeding is usually the first symptom of debut in affected patients; it is also possible to present intermenstrual bleeding in premenopausal women and abnormal uterine bleeding in postmenopausal women [6].

Immunohistochemistry is an important tool because it is used to confirm tissue of origin and distinguish metastatic from primary tumor [11]. A study where 57 cases of metastatic breast cancer were analyzed showed the marker GATA-3 as the highest frequency of expression, being in 82% of the cases [12]. This showed the marker GATA-3 as a sensitive marker for identifying metastatic breast cancer, with superior sensitive marker compared to other ones as mammaglobin.

This case report and literature review remarks the importance of complete approach and diagnosis in patients with abnormal uterine bleeding, particularly in presence of breast cancer history, as our patient. It is also important to be noted that breast cancer patients under hormonal treatments like aromatase inhibitors or tamoxifen may have primary endometrial cancer as well as uterine metastases [13].

It is important to make a precise diagnosis with clinical history and immunohistochemistry to differentiate a metastatic breast tumor from a primary genital neoplasm, since the treatments and prognosis are completely different [14]; a primary uterine neoplasm can be surgically resected, while in uterine metastases surgical intervention does not appear to be indicated and systemic chemotherapy would be probably a better noninvasive option for treatment [14]. However, because of the limited number of case reports, there is not enough data about the prognosis. more cases and more studies will be needed to improve our knowledge about the best treatment and precise prognosis for those patients [15].

## References

[1] Hara F., Kiyoto S., Takabatake D. (2010). Endometrial metastasis from breast cancer during adjuvant endocrine therapy. *Case Reports in Oncology*.

[2] Fichman D. M., Lossio J. J., Wajngarten I. I. (1977). Evaluation of cutting efficiency of manual cutting instruments. II. Natural teeth. *Revista da Faculdade de Odontologia da Universidade de Sao Paulo*.

[3] Cift T., Aslan B., Bulut B., Ilvan S. (2016). Unusual uterine metastasis of invasive ductal carcinoma: a case report. *Turkish Journal of Obstetrics and Gynecology*.

[4] Razia S., Nakayama K., Tsukao M., Nakamura K., Ishikawa M., Ishibashi T. (2017). Metastasis of breast cancer to an endometrial polyp, the cervix and a leiomyoma: a case report and review of the literature. *Oncology Letters*.

[5] Brunicardi F. C., Andersen D. K., Billiar T. R., Dunn D. L., Hunter J. G., Matthews J. B. (2015). *Schwartz's Principles of Surgery*.

[6] Bezpalko K., Mohamed M. A., Mercer L., McCann M., Elghawy K., Wilson K. (2015). Concomitant endometrial and gallbladder metastasis in advanced multiple metastatic invasive lobular carcinoma of the breast: a rare case report. *International Journal of Surgery Case Reports*.

[7] Arpino G., Bardou V. J., Clark G. M., Elledge R. M. (2004). Infiltrating lobular carcinoma of the breast: tumor characteristics and clinical outcome. *Breast Cancer Research*.

[8] Ustaalioglu B. B. O., Bilici A., Seker M. (2009). Metastasis of lobular breast carcinoma to the uterus in a patient under anastrozole therapy. *Onkologie*.

[9] Yazigi R., Sandstad J., Munoz A. K. (1988). Breast cancer metastasizing to the uterine cervix. *Cancer*.

[10] Kowalski P. J., Rubin M. A., Kleer C. G. (2003). E-cadherin expression in primary carcinomas of the breast and its distant metastases. *Breast Cancer Research*.

[11] Zaha D. C. (2014). Significance of immunohistochemistry in breast cancer. *World Journal of Clinical Oncology*.

[12] Tozbikian G. H., Zynger D. L. (2018). A combination of GATA3 and SOX10 is useful for the diagnosis of metastatic triple negative breast cancer. *Human Pathology*.

[13] Groß S., De Waal J., Neteler J., Debus G. (2011). Uterocervical metastasis of an invasive ductal breast carcinoma: a case report. *Geburtshilfe Frauenheilkd*.

[14] Huo Z., Gao Y., Zuo W., Zheng G., Kong R. (2015). Metastases of basal-like breast invasive ductal carcinoma to the endometrium: a case report and review of the literature. *Thoracic Cancer*.

[15] Karvouni E., Papakonstantinou K., Dimopoulou C. (2009). Abnormal uterine bleeding as a presentation of metastatic breast disease in a patient with advanced breast cancer. *Archives of Gynecology and Obstetrics*.

